# Effect of the sodium‐glucose cotransporter‐2 inhibitor, DWP16001, as an add‐on therapy to insulin for diabetic dogs: A pilot study

**DOI:** 10.1002/vms3.1454

**Published:** 2024-04-30

**Authors:** Ju‐Hyun An, Han‐Sol Choi, Ji‐Soo Choi, Hyun‐Woo Lim, Wan Huh, Ye‐In Oh, Joon Seok Park, Jumi Han, Soo Lim, Chae‐Young Lim, Tae‐Hee Kim, Jae‐Bong Moon, Hwa‐Young Youn

**Affiliations:** ^1^ Department of Veterinary Emergency and Critical Care Medicine and Institute of Veterinary Science College of Veterinary Medicine Kangwon National University Chuncheon‐si Republic of Korea; ^2^ Laboratory of Veterinary Internal Medicine Department of Veterinary Clinical Science College of Veterinary Medicine Seoul National University Gwanak‐gu Seoul Republic of Korea; ^3^ Daewoong Pharmaceutical Life Science Institute Yongin Gyeonggi‐do Republic of Korea; ^4^ Department of Veterinary Internal Medicine College of Veterinary Medicine Kyungpook National University Daegu Republic of Korea; ^5^ Department of Internal Medicine, Seoul National University College of Medicine Seoul National University Bundang Hospital Seongnam Republic of Korea; ^6^ Nowon 24 hours N Animal Medical Center Nowon‐gu Seoul Republic of Korea; ^7^ Daewoong pet Boneunsa‐ro Gangnam‐gu Seoul Republic of Korea

**Keywords:** dogs, DWP16001, diabetes mellitus, sodium‐glucose cotransporter‐2 inhibitor

## Abstract

**Background:**

Sodium‐glucose cotransporter‐2 (SGLT2) inhibitors are a novel class of anti‐hyperglycaemic agents.

**Objective:**

This study aimed to evaluate the safety and the adjuvant glycaemic control effect of an SGLT2 inhibitor, DWP16001, in diabetic dogs receiving insulin treatment.

**Methods:**

Nineteen diabetic dogs receiving insulin treatment (NPH, porcine lente and glargine insulin) were divided into two groups according to dosing frequency: DWP TOD group (*n* = 10) and DWP SID group (*n* = 9). In the DWP TOD group, 0.025 mg/kg of DWP16001 was administered once every 3 days, whereas, in the DWP SID group, 0.025 mg/kg of DWP16001 was administered once a day. Food intake was maintained during the trial period. Hypoglycaemia, ketoacidosis or unexpected life‐threatening reactions were assessed as adverse effects before and after DWP16001 administration. We compared insulin requirement reduction and blood glucose level control between two groups.

**Results:**

No specific adverse effects were observed during the clinical trial, and haematological parameter remained unchanged. Moreover, the fasting glucose levels and daily insulin dose in the DWP TOD group were lower than the pre‐administration values, but not significantly different for 8 weeks. Systolic blood pressure, fructosamine and insulin dose decreased significantly in the DWP SID group compared to the DWP TOD group at 8 weeks (*p* < 0.05) without affecting food consumption. Among these patients, 10 patients were monitored while receiving DWP16001 for 12 months (DWP TOD group *n* = 5, DWP SID group *n* = 5). The fasting glucose and fructosamine levels and daily insulin dose were reduced in both groups at 12 months compared with those before receiving DWP16001.

**Conclusion:**

When DWP16001, an SGLT2 inhibitor, was supplied to dogs with type 1 diabetes, no adverse effects were observed, and it was confirmed that the administered insulin dose can be reduced in controlling blood glucose.

## Graphical abstract

Administration of the SGLT2 inhibitor, DWP16001, can be an adjunctive method for managing diabetes in terms of glycaemic control without adverse effects in diabetic dogs receiving insulin therapy. This study will serve as the primary basis for the application of SGLT2 inhibitors in dogs with insulin‐dependent diabetes mellitus.

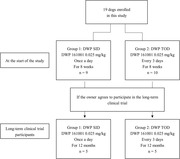

## 1 INTRODUCTION

Diabetes mellitus (DM) is a heterogeneous group of diseases with multiple aetiologies characterized by hyperglycaemia resulting from inadequate insulin secretion, inadequate insulin action or both (Niessen et al., 2022). Insulin therapy remains the treatment of choice for diabetic dogs; however, achieving good glycaemic control is often difficult as multiple insulin injections are required daily and the dosage is limited due to the possibility of hypoglycaemia (Davison et al., 2005; Hardin et al., 2015). In humans, research is actively underway to evaluate the safety and efficacy of adjuvant treatments to non‐insulin drugs, such as sodium‐glucose cotransporter‐2 (SGLT2) inhibitors and GLP‐1 receptor agonists, in diabetic patients with inadequate glycaemic control (Castellana et al., 2019). However, there is still an unmet need for adjunctive treatments other than insulin therapy to address the twin challenges of managing hyperglycaemia and hypoglycaemia in diabetic dogs. It should be noted that as the addition of non‐insulin drugs in type 1 diabetes in humans is a promising strategy for improving glycaemic control in patients with diabetes (Baștan & Börkü, 2010; Frandsen et al., 2016; Wright & Hirsch, 2019), it may also be a possibility in dogs.

SGLT2 inhibitors are a novel class of anti‐hyperglycaemic agents. SGLT2 inhibitors reduce glucose reabsorption at the proximal nephron, leading to increased glucose excretion through a mechanism that is independent of insulin (Evans et al., 2020). The safety and efficacy of SGLT2 inhibitors in the treatment of type 2 DM (T2DM) have been established in previous human studies (Haneda et al., 2016), and human studies are currently being conducted to explore their potential application in the treatment of type 1 DM (T1DM) (El Masri et al., 2018; Yamada et al., 2018). Recent human studies on the treatment of T1DM with a combination of an SGLT2 inhibitor and insulin have shown that SGLT2 inhibitors assist in controlling the glycaemic levels and reduce the insulin requirements (Evans et al., 2020). The adverse effects of SGLT2 inhibitors in humans are usually mild and related to glucosuria and osmotic diuresis. The incidence of serious adverse effects, such as ketoacidosis and severe genitourinary infections, is rare (Chesterman & Thynne, 2020). However, in veterinary medicine, no studies have confirmed the safety and efficacy of the combination of an SGLT2 inhibitor and insulin in the treatment of diabetic dogs.

Therefore, in this study, we aimed to evaluate the safety and efficacy of DWP16001 (Enavogliflozin, Daewoong Pharmaceutical Co. Ltd.), an SGLT2 inhibitor, by evaluating clinical symptoms, glycaemic control and reduction of insulin requirements in insulin‐dependent diabetic dogs receiving insulin treatment.

## 2 MATERIALS AND METHODS

### 2.1 Study design

This study was conducted under the hypothesis that the combined administration of insulin therapy and DWP16001, one of the SGLT2 inhibitors, to dogs with naturally occurring insulin‐dependent diabetes could help control blood glucose, without life‐threatening adverse effects.

We determined the number of samples per each group to investigate our major hypothesis, the fructosamine in the dog with insulin treatment might be higher than that of the dog with insulin and SLGT‐2 inhibitors treatment or not. Our study estimated the minimum number of samples in each group considering our statistical approach, the student *t* test. The significant level and statistical power of the statistical test were 0.05 (alpha) and 0.8 (beta), respectively. We expected the mean of the fructosamine in the dog with insulin treatment might be around the reference value of the fructosamine under ‘Fair control’ (450–500 µmol/L) and the fructosamine in the dog with insulin and SLGT‐2 inhibitors treatment would be under ‘Excellent control’ (350–400 µmol/L). Under the expected standard deviation of the fructosamine value as 80 µmol/L, the effective size (d) of this hypothesis was determined to 1.25. With those values, the minimum number of samples was determined 9 (*n* = 8.678197) in each group using the ‘prw’ package on R (Version 4.3.1). Based on this, the study was designed to have at least nine animals in each of the two groups.

The procedures performed in this study were approved by the Institutional Animal Care and Use Committee of Seoul National University (SNU) (SNU; protocol no. SNU‐200921‐5). As client‐owned animals were included in this study, all procedures were conducted over a period of 1 year after receiving consent from the owner.

This study was conducted as a prospective proof‐of‐concept open‐label test. To exclude variables that could affect the clinical trial, dogs receiving concomitant medications that could affect the blood pressure, renal function and glycaemic levels were excluded. Therefore, patients who had received diuretics within 14 days before the administration of DWP16001 were excluded from the study. Only patients who had maintained a stable insulin dose for 1 month, had blood ketones in the normal range as measured by a ketone meter, which measures blood beta‐hydroxybutyrate levels, and had no confirmed urine ketones as measured by a dipstick were included in this test. DWP16001 was administered along with breakfast and the patient had access to water at all times during the testing period. The occurrence of adverse effects, including appetite, vitality, stool, vomiting, water intake and urine excretion, was assessed by phone 1 week after initiating drug administration. The patients visited the veterinary hospital on day 1, day 14, day 28, day 56, 3 months, 6 months, 9 months and 12 months according to the date of receiving DWP16001. In addition to a general physical examination, including the assessment of weight and blood pressure, other tests to evaluate the complete blood count, serum chemistry, urinalysis, fasting blood glucose, blood fructosamine, ketones, LDH levels and insulin requirements were also performed.

In the case of severe abnormalities, such as hypoglycaemia and ketoacidosis or unexpected life‐threatening reactions (adverse reaction/adverse drug reaction), the owners were instructed to contact the clinical investigator and visit the veterinary hospital for appropriate treatment. If the clinical investigator determined that the treatment cannot be continued during the visit, the treatment was discontinued, and the dog was excluded from the clinical trial after consultation with the owner.

### 2.2 Drug information

DWP16001[(2S,3R,4R,5S,6R)‐2‐(7‐chloro‐6‐(4‐cyclopropylbenzyl)‐2,3‐dihydrobenzofuran‐4‐yl)‐6‐(hydroxymethyl)tetrahydro‐2H‐pyran‐3,4,5‐triol] (Kim et al., 2020), an SGLT2 inhibitor developed by Daewoong Pharmaceutical, has shown promising results in phase 2 clinical trials on humans (registration number NCT04014023). Similar results were observed in a recently completed phase 3 clinical trials on humans. Based on these results, DWP16001 has received final approval from the Ministry of Food and Drug Safety (KFDA) (registration number NCT04632862) (Han et al., 2023).

### 2.3 Canine patients

Diabetic dogs unable to achieve glycaemic control with insulin alone can be included in the study if they meet the following criteria: small dogs (<10 kg) under 16 years of age, medium dogs (11–22 kg) under 13 years of age, nadir blood glucose level of at least 200 mg/dL and blood ketone levels of <0.6 mmol/L. In addition, before taking DWP16001, the prescribed insulin dose must not have changed for at least 1 month. The principal exclusion criteria were as follows: dogs with chronic kidney disease (IRIS stage >2), dogs evaluated for hypotension, dogs receiving diuretics, dogs with proven pregnancy and dogs that had taken prescription drugs or over‐the‐counter drugs within 14 days before the start of the test (administration of the test product) and whose drugs were judged by the investigator to affect the test or affect safety were excluded. By applying the above criteria, the study was ultimately conducted on 19 dogs out of 38 dogs who wished to participate in the study (Shiel & Mooney, 2022).

### 2.4 Procedures

Participants were randomly divided into two groups and took the drug at different frequencies, and both groups received the drug continuously throughout the study period. The DWP TOD group received 0.025 mg/kg of DWP16001 once every 3 days, whereas the DWP SID group received 0.025 mg/kg of DWP16001 once a day (Choi et al., 2020; Lee et al., 2023).

The selected participating dogs were observed for changes in fasting glucose levels, fructosamine, body weight, mean daily bolus dose of insulin from the baseline to week 8. Additionally, the glycaemic levels were monitored for 1 year in dogs whose owners consented to the long‐term administration of the drug.

The owners of participating dogs received information about adverse events related to the use of antihyperglycaemic agents, such as severe hypoglycaemia, ketoacidosis, bacterial cystitis and dehydration, focusing on the adverse effects identified when SGLT‐2 inhibitors were administered to humans in previous studies (Garofalo et al., 2019; Halimi & Vergès, 2014). Hypoglycaemia is generally defined as low blood glucose levels in dogs, where the blood glucose level is less than 3.3 mmol/L (60 mg/dL). In this study, considering in‐home monitoring for 1‐year, severe hypoglycaemia was defined based on the following clinical signs. Clinical signs included changes in mental status and behaviour, seizures, syncope, muscle spasms/contractions, somnolence, exercise intolerance, muscle tremors, collapse, ataxia, weakness and visual disturbances (Idowu & Heading, 2018). Diabetic ketoacidosis (DKA) was diagnosed based on anion gap metabolic acidosis due to excessive ketone production without an alternative cause. If it became impossible for the dogs to continue this clinical trial, the treatment was terminated after consulting with the investigator. Safety information, including clinical, laboratory and adverse events data, was collected at each visit. In addition, the owner and veterinarian completed questionnaires regarding the appetite, stool, active vomiting, water intake and urine excretion at the time of examination and evaluated the abnormal condition after receiving DWP16001. To summarize briefly, it is as follows: appetite (0. same, 1. increased, 2. decreased, 3. none); vitality (0. same, 1. increase, 2. decrease, 3. none); faeces (0. hard, 1. normal stool, 2. soft stool, 3. diarrhoea); vomiting (0. normal, 1. once a week, 2. 2–3 times a week, 3. more than 3 times a week); water intake (0. same, 1. increase, 2. decrease); urination (0. same, 1. increased, 2. decreased).

### 2.5 Statistical analyses

Among patients with diabetes who received DWP16001 for 8 weeks, only dogs who agreed to long‐term use of DWP16001 were monitored for 1 year after receiving DWP16001. Differences between the two groups before the administration of DWP16001 and at the time of examination were compared within group. Differences between the two groups at the time of the survey were also compared. Normality was assessed using the Shapiro–Wilk test. Homogeneity of variance was assessed using Levene's test. Differences within the groups were compared using the student's paired *t* test or Wilcoxon rank test. Additionally, the difference between the groups after drug administration was assessed using a mixed analysis of variance test. Statistical analyses were performed using the R programming language (https://cran.r‐project.org/) and GraphPad Prism version 7 (GraphPad Software, Inc.). A *p*‐value of <0.05 was considered statistically significant.

## 3 RESULTS

### 3.1 Patient characteristics

Nineteen diabetic dogs receiving insulin (NPH, glargine or porcine lente insulin) once or twice daily who met the inclusion criteria were divided into two groups (DWP TOD and DWP SID groups). The breed, sex, age and body condition score (BCS) of the participating dogs were taken into consideration when allocating the dogs to groups and were allocated as evenly as possible. The DWP TOD group included four Poodles, two Schnauzers, one Golden retriever, one Maltese, one Spitz and one Mongrel. Among them, six were castrated males, three were spayed females, and one was an intact female. Their ages ranged from 5 to 16 years old, with a mean age of 12 years. The BCS ranged from 4 to 6, with a mean score of 4. The mean systolic blood pressure (mmHg) was 141.8 ± 20.5, and the mean insulin dose was 1.6 ± 0.4 (IU/kg/day).

The DWP SID group included five Maltese dogs, one Poodle, one Yorkshire Terrier, one Mongrel and one Samoyed. Among them, six were castrated males, two were spayed females, and one was an intact female. Their ages ranged from 8 to 15 years old, with a mean age of 10.5. The BCS ranged from 3 to 7, with a mean score of 5. The mean systolic blood pressure (mmHg) was 157.3 ± 24.9, and the mean insulin dose was 2.0 ± 0.8 (IU/kg/day) (Table 1).

**TABLE 1:** Population characteristics of the diabetic dogs enrolled in the clinical trial using DWP16001.

		Group	
Variables	Reference ranges	DWP TOD (*n* = 10)	DWP SID (*n* = 9)
Breed	**NA**	Golden retriever (1), Maltese (1), Schnauzer (2), Spitz (1), Poodle (4), Mongrel (1)	Maltese (5), Poodles (1), Yorkshire terrier (1), Mongrel (1), Samoyed (1)
Sex	**NA**	CM (6), SF (3), F (1)	CM (6), SF (2), F (1)
Age, years	**NA**	12 (5–16)	10.5 (8–15)
BCS	**NA**	4 (4–6)	5 (3–7)
Systolic blood pressure (mmHg)	**<140**	141.8 ± 20.5	157.3 ± 24.9
Insulin dose (IU/kg/day)	**NA**	1.6 ± 0.4	2.0 ± 0.8

*Note*: Breed, sex, age, BCS, systolic blood pressure and insulin dose of diabetic dogs participating in this study. The data are expressed as median (range) for age and BCS, and as mean ± standard deviation for systolic blood pressure and insulin dose.

Abbreviations: BCS, body condition score; CM, castrated male; DWP SID, group that received 0.025 mg/kg of DWP16001 once a day; DWP TOD, group that received 0.025 mg/kg of DWP16001 once every 3 days; F, female; M, male; SF, spayed female.

**FIGURE 1:**
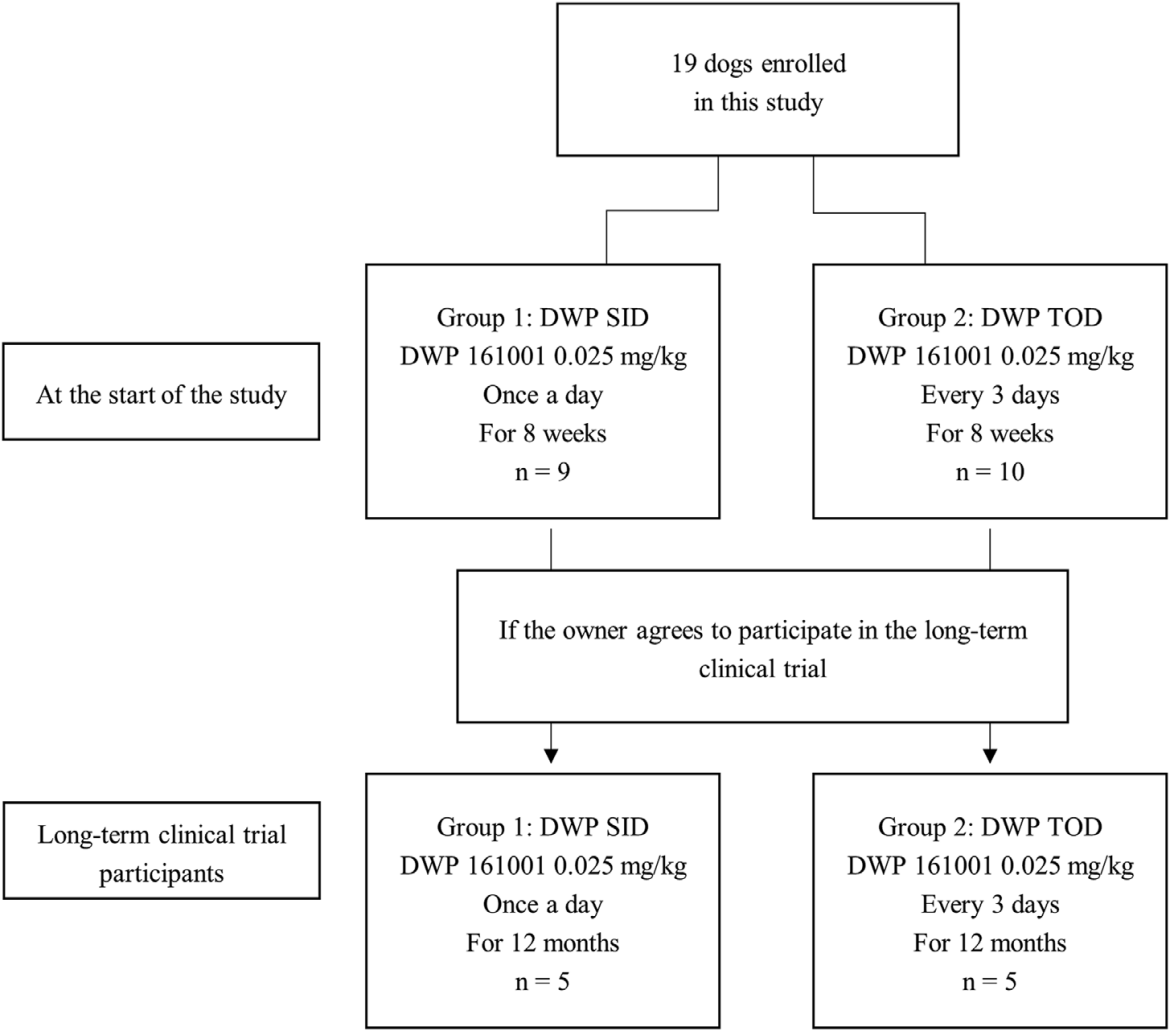
Flow diagram of changes in the group composition of dogs enrolled in this study. In this study, 19 dogs were divided into two groups according to the dosing frequency. After 8 weeks, the clinical trial was continued for a period of 1 year in 10 dogs after receiving consent from their owners.

Among the 19 patients who participated in the test, 9 dropped out; thus, 10 patients were monitored for a duration of 1 year. A summary of this is shown in Figure 1.

**FIGURE 2:**
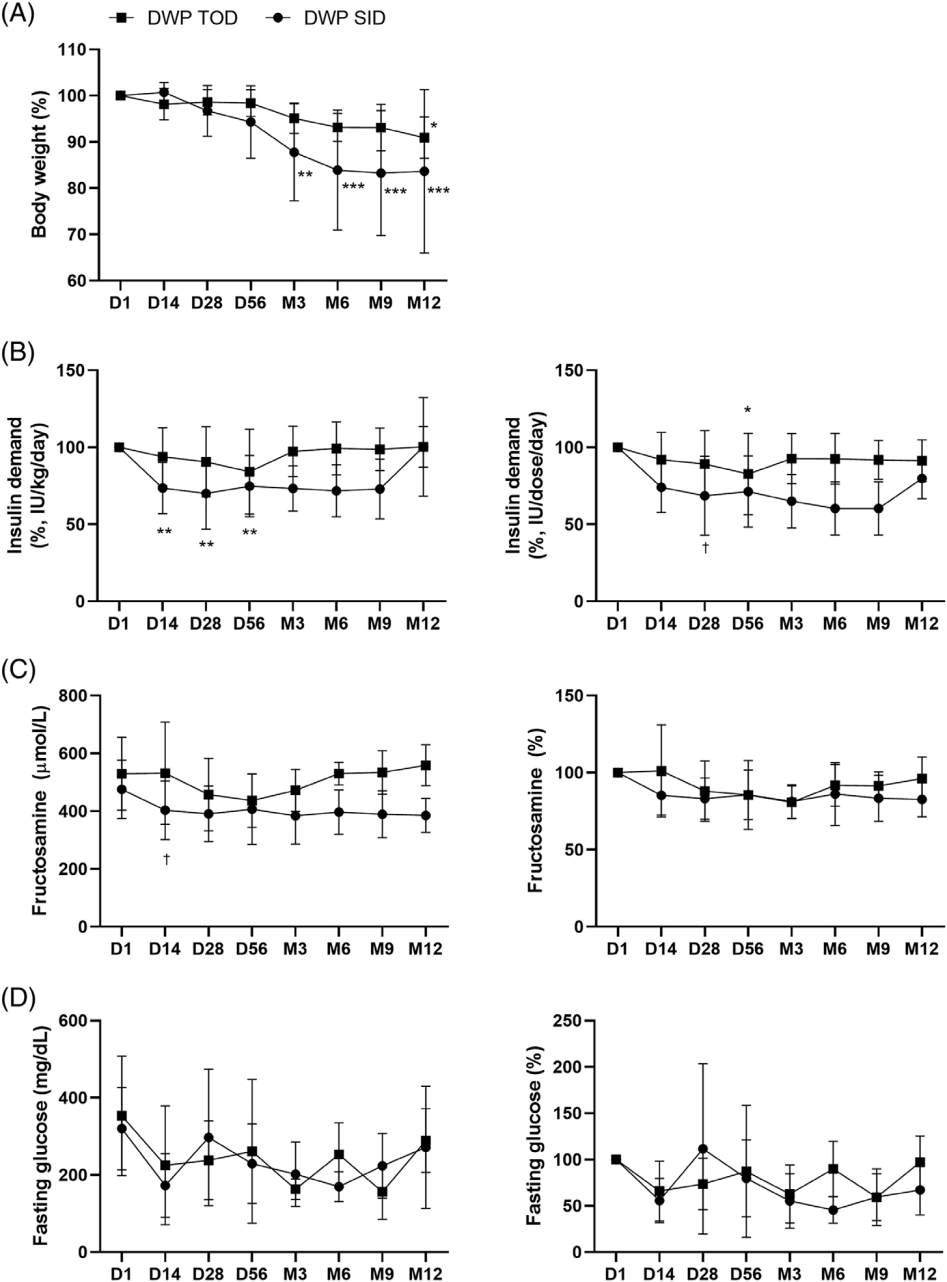
Effect of DWP16001 on the changes in body weight, insulin demand, fructosamine concentration and fasting glucose levels in the diabetic dogs who participated in the clinical trial for 1 year (A) changes in body weight percentage, (B) changes in insulin dose and percentage, (C) changes in blood fructosamine level and percentage, (D) changes in the fasting blood glucose level and percentage. The results are presented as mean ± standard deviation (^*^ *p* < 0.05 ^**^ *p* < 0.01 and ^***^ *p* < 0.001, comparison with D1 within each group; ^†^ *p* < 0.05, Comparison between groups at each time point). DWP TOD, group that received 0.025 mg/kg of DWP16001 once every 3 days; DWP SID, group that received 0.025 mg/kg of DWP16001 once a day.

Significant weight loss was observed after 3 months of receiving the drug in the SID group compared with that before the administration (*p *< 0.01). In both groups, significant weight loss was observed after receiving the drug for 12 months compared with that before the administration (*p *< 0.001) (Figure 2A). The total insulin requirement for glycaemic maintenance decreased in both groups. In particular, a significant reduction was observed after 2 months of receiving the drug in the DWP SID (*p* < 0.01) (Figure 2B). Although not significant in both groups, a decreasing trend was observed in the fructosamine and fasting glucose levels (Figure 2C,D).

### 3.2 Assessment of blood ketone after treatment with DWP16001

**FIGURE 3:**
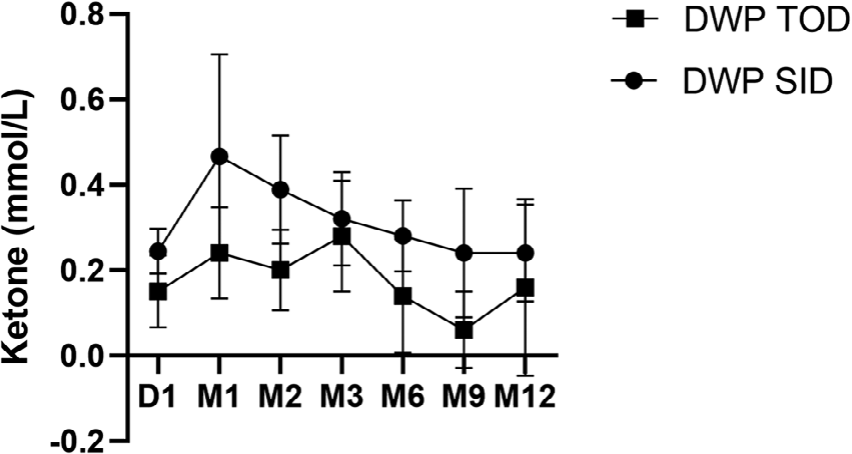
Effect of DWP16001 on the changes in blood ketone level in diabetes dogs at each study time point. Change in blood ketone level in each group. Data are expressed as mean ± standard deviation. DWP TOD, group that received 0.025 mg/kg of DWP16001 once every 3 days; DWP SID, group that received 0.025 mg/kg of DWP16001 once a day.

Changes in blood ketone levels were assessed by measuring serum or whole blood beta‐hydroxybutyrate 12 months after the administration of DWP16001. No significant differences in blood ketone levels were observed between the two groups before administration, and no significant changes in blood ketone levels were observed in either group for 12 months after administration (Figure 3).

### 3.3 Assessment of haematological findings and serum biochemistry after treatment with DWP16001

No significant difference was observed in the haematological findings in either group over the 12‐month test period. In addition, no significant change was observed in the serum chemistry test results of either group before or after taking the drug (Table 2).

**TABLE 2:** Changes in complete blood count and serum biochemistry in diabetic dogs enrolled in the clinical trial using DWP16001 for 12 months.

		DWP TOD group			DWP SID group		
		Basal	D56	12M	Basal	D56	12M
Parameter	Reference range	(*n* = 10)	(*n* = 10)	(*n* = 5)	(*n* = 9)	(*n* = 9)	(*n* = 5)
WBC	**5.2–13.9 k/µL**	10.13 ± 3.93	7.94 ± 1.80	7.20 ± 0.81	10.17 ± 4.13	9.79 ± 3.35	8.96 ± 2.81
RBC	**5.7–8.8 M/µL**	6.04 ± 1.29	6.28 ± 0.96	6.58 ± 0.40	7.05 ± 0.51	6.54 ± 0.89	6.59 ± 0.25
HCT	**37.1%–57%**	42.98 ± 9.48	44.42 ± 7.18	48.50 ± 3.37	50.71 ± 4.65	47.49 ± 3.33	51.16 ± 4.48
Hb	**12.9–18.4 g/dL**	14.56 ± 3.40	13.61 ± 4.54	15.80 ± 0.95	16.56 ± 1.24	16.22 ± 1.68	16.28 ± 1.01
PLT	**143.3–400 k/µL**	287.00 ± 112.99	321.00 ± 65.73	341.80 ± 66.63	352.56 ± 134.40	382.78 ± 117.34	361.20 ± 129.90
TP	**4.9–7.2 g/dL**	6.49 ± 0.52	6.35 ± 0.56	6.55 ± 0.51	6.39 ± 0.79	6.24 ± 0.60	6.22 ± 0.67
ALB	**2.3–3.9 g/dL**	2.77 ± 0.45	2.80 ± 0.33	2.99 ± 0.34	2.95 ± 0.48	3.04 ± 0.34	2.83 ± 0.51
ALP	**20–155 U/L**	1050.40 ± 651.12	607.80 ± 461.38	587.20 ± 155.98	1604.67 ± 1262.93	1507.44 ± 2448.94	1673.20 ± 1453.49
AST	**10–37 U/L**	41.61 ± 16.79	37.62 ± 17.39	33.86 ± 9.12	43.10 ± 24.22	49.31 ± 35.47	68.60 ± 50.78
ALT	**3–50 U/L**	41.41 ± 21.79	31.13 ± 12.04	43.20 ± 20.32	88.99 ± 51.93	69.41 ± 54.67	119.48 ± 52.96
GGT	**4–25 U/L**	13.35 ± 7.90	6.64 ± 2.77	6.84 ± 3.73	28.27 ± 24.18	26.02 ± 29.87	17.98 ± 17.78
Tbil	**0.1–0.7 mg/dL**	0.20 ± 0.16	0.16 ± 0.08	0.17 ± 0.11	0.14 ± 0.02	0.19 ± 0.07	0.14±0.03
TC	**127–340 mg/dL**	377.48 ± 159.55	327.64 ± 114.68	547.00 ± 276.39	340.43 ± 218.52	253.92 ± 142.51	289.70 ± 177.18
TG	**21–116 mg/dL**	529.74 ± 846.90	384.91 ± 425.34	814.52 ± 975.01	383.92 ± 804.14	478.51 ± 921.32	316.48 ± 272.85
CK	**25–167 U/L**	205.58 ± 116.22	206.43 ± 175.39	169.22 ± 54.75	138.29 ± 97.01	124.48 ± 27.28	92.20 ± 10.07
LDH	**65–269 U/L**	435.63 ± 412.05	444.45 ± 584.70	245.26 ± 94.49	363.79 ± 418.49	215.16 ± 88.08	108.58 ± 44.49
BUN	**5–30 mg/dL**	21.30 ± 11.73	22.50 ± 11.70	21.23 ± 6.38	14.34 ± 5.33	20.80 ± 7.13	25.09 ± 12.58
CREA	**0.5–1.5 mg/dL**	0.86 ± 0.21	0.88 ± 0.21	0.78 ± 0.29	0.64 ± 0.12	0.64 ± 0.15	0.83 ± 0.18
Amylase	**388–1007 U/L**	752.45 ± 255.21	691.15 ± 299.19	888.08 ± 324.90	877.77 ± 355.44	713.33 ± 278.88	1068.58 ± 425.36
Lipase	**5–90 U/L**	176.10 ± 145.22	197.30 ± 230.09	230.80 ± 132.39	281.89 ± 328.09	213.56 ± 233.10	238.40 ± 179.10
Na^+^	**139–149 mmol/L**	145.91 ± 3.18	147.06 ± 5.93	146.76 ± 1.16	145.49 ± 3.75	148.91 ± 3.29	148.02 ± 4.42
K^+^	**3.5–5.2 mmol/L**	4.79 ± 0.60	4.95 ± 0.37	4.78 ± 0.35	5.13 ± 0.61	4.94 ± 0.65	4.71 ± 0.45
Cl^−^	**104–118 mmol/L**	110.64 ± 2.97	111.34 ± 5.92	109.26 ± 1.29	111.01 ± 2.68	112.84 ± 3.09	111.60 ± 3.51
Ca	**8.0–11.7 mg/dL**	9.90 ± 1.29	9.82 ± 0.76	10.46 ± 1.15	9.59 ± 0.80	10.12 ± 0.89	10.02 ± 0.69
P	**2.4–6.4 mg/dL**	3.57 ± 0.92	3.91 ± 0.61	3.50 ± 1.73	3.73 ± 1.15	4.21 ± 1.40	4.08 ± 1.21
SDMA	**≤18 µg/dL**	8.80 ± 2.94	9.20 ± 5.51	6.60 ± 1.67	8.56 ± 2.60	7.67 ± 1.32	8.80 ± 3.96

*Note*: Data are expressed as mean ± standard deviation.

Abbreviations: ALB, albumin; ALP, alkaline phosphatase; ALT, alanine aminotransferase; AST, aspartate aminotransferase; BUN, blood urea nitrogen; Ca, calcium; CK, creatinine kinase; Cl^−^, chloride iron; CREA, creatinine; GGT, gamma glutamyl transferase; Hb, haemoglobin; HCT, haematocrit; K^+^, potassium ion; LDH, lactate dehydrogenase; Na^+^, sodium ion; P, phosphorus; PLT, platelet; RBC, red blood cell; SDMA, symmetric dimethylarginine; Tbil, total bilirubin; TC, total cholesterol; TG, triacylglycerol; TP, total protein; WBC, white blood cell.

### 3.4 Adverse reactions

As a result of checking the appetite, vitality, faeces, vomiting, water intake and urination of the two groups before and after drug administration during the study period, only one animal in the once‐daily administration group showed an increase in appetite 12 months after administration, and the remaining patients showed no obvious abnormal signs. Additionally, no severe hypoglycaemia that could lead to clinical symptoms was identified in any of the patients participating in the clinical trial, and no ketoacidosis or dehydration was observed. Two patients in the DWP SID group had cystitis when they visited a veterinary hospital at 12 months; however, the clinical symptoms related to cystitis improved after antibiotic treatment.

## 4 DISCUSSION

The purpose of this study is to primarily evaluate the safety of insulin and DWP16001, a hypoglycaemic agent, in diabetic dogs when administered together to insulin‐dependent diabetic dogs, and secondarily to evaluate the effectiveness.

DWP16001, an SGLT2 inhibitor, developed by Daewoong Pharmaceutical, has recently completed phase 3 clinical trials on humans. It has received final approval from the KFDA (registration number NCT04632862). According to pharmacokinetic and pharmacodynamic studies conducted in mice, DWP16001 had a higher renal distribution, greater effect and greater persistence than those of dapagliflozin and ipragliflozin (Choi et al., 2020). In addition, a recent study on the use of DWP16001 in humans with diabetes suggested that DWP16001 can be used as an alternative to dapagliflozin. These findings suggest that DWP16001 can be an excellent alternative to existing SGLT‐2 inhibitors in human T2DM (Han et al., 2023). However, in order to apply it as a therapeutic option in veterinary medicine, its therapeutic effect must be evaluated in dogs and cats with naturally occurring diabetes.

In this study, DWP16001 controls blood glucose level with reducing the insulin demand without inducing DKA in diabetic dogs. These findings are similar to those reported in human studies that involved the administration of SGLT2 inhibitors to patients with DM. Previous studies have shown that SGLT2 inhibitors significantly improved the levels of major glycaemic control parameters (fructosamine, fasting plasma glucose, blood pressure and insulin dose) in humans (Chao, 2014). In addition, it was observed that the administration of DWP16001 lowered the body weight in diabetic dogs. If the body's use of glucose is inadequate in diabetic patients, weight loss may occur due to a decrease in intracellular glucose uptake and osmotic dehydration due to urinary glucose. However, in the case of the patients who participated in this test, diabetic ketosis did not occur as confirmed through questionnaires and physical examination, and dehydration was not confirmed. It is believed that pathological weight loss due to diabetes was not observed. In contrast, previous studies in humans have shown that one of the side effects of insulin therapy is weight gain (Russell‐Jones & Khan, 2007). Insulin‐related weight gain occurs due to the anabolic effects of insulin, increased appetite and reduced glycosuria (Longo et al., 2019). Therefore, the use of the hypoglycaemic drug metformin in combination with insulin is recommended to limit weight gain in patients with diabetes. Other newly developed oral therapies and insulin analogues may also aid weight control (Heller, 2004). A study reported weight loss in obese dogs after the administration of DWP16001 (Rhee et al., 2022); however, no study has evaluated the weight change in dogs when DWP16001 is used in combination with insulin treatment. Although the exact relationship between blood glucose and blood pressure remains unknown, the spectrum of vascular complications associated with DM in dogs is similar to that observed in humans and includes systemic hypertension. Through this study, weight loss and changes in blood pressure were confirmed after the administration of DWP16001 in diabetic dogs undergoing insulin treatment, and it aid in predicting the patient's condition during DWP16001 treatment. However, it is necessary to evaluate weight loss and subsequent side effects when DWP16001 is applied to a larger number of diabetic dogs.

DWP16001 was applied to dogs with insulin‐dependent DM in this study. Previous studies on SGLT2 inhibitor administration in humans have confirmed the safety and efficacy of SGLT2 inhibitors for the treatment of T2DM; thus, SGLT2 inhibitors are primarily recommended for the treatment of patients with T2DM. Recent human studies have reported that SGLT2 inhibitors effectively control blood glucose levels and reduce insulin requirements without serious adverse effects in T1DM when administered in combination with insulin (Yang et al., 2017). However, no veterinary studies have systematically confirmed the efficacy and safety of a combination of SGLT2 inhibitors and insulin in dogs with T1DM. Thus, this study is important in that it evaluated the effectiveness and safety of DWP16001 in dogs with naturally occurring T1DM. Additionally, it should be noted that the administration of DWP16001 in diabetic dogs receiving insulin did not show any serious adverse health effects.

DKA is a medical emergency characterized by hyperglycaemia (blood glucose >250 mg/dL), metabolic acidosis (arterial pH <7.3 and serum bicarbonate level <18 mEq/L) and ketosis. Diabetes acidosis occurring in patients receiving hypoglycaemic drugs with blood glucose levels <200 mg/dL is defined as euglycaemic DKA (Ogawa & Sakaguchi, 2016). The mechanism by which SGLT2 inhibitors induce euglycaemic DKA remains unclear (Chow et al., 2023). Although the SGLT2 receptor is predominantly found in the proximal tubule of the nephron, which does not readily explain its potential role in DKA, recent research has revealed its presence in the alpha cells of the pancreas responsible for glucagon secretion. This direct activation of glucagon secretion, combined with the persistent glycosuria induced by SGLT2 inhibitors, leads to a catabolic state. This catabolic state indirectly triggers the release of glucagon and catecholamines. Additionally, these inhibitors have been found to reduce insulin secretion by pancreatic beta‐cells. The resulting imbalance between insulin and counterregulatory hormones can promote lipolysis and ketogenesis, which can be further exacerbated by factors such as volume depletion or an excess of catecholamines, such as concurrent illness or reduced food and water intake. Lactic acidosis is a common finding in DKA. Anaerobic glycolysis due to inadequate tissue perfusion and oxygenation as well as metabolic disorders associated with DKA can contribute to elevated lactate levels (Blau et al., 2017). In our study, the ketone levels tended to increase in diabetic dogs receiving the hypoglycaemic drug once a day; however, it increased within the range of normal ketone levels. Moreover, acidosis and elevated lactate levels were not observed in the venous blood analysis. No significant adverse effects related to metabolic acidosis were identified in this study. However, as adverse effects have been reported in previous studies, it is recommended to administer the drug after close monitoring of metabolic acidosis in human patients with DM (Ogawa & Sakaguchi, 2016). Additionally, no adverse effects on kidney function, lipid metabolism and hydration were observed in this study.

Some studies have shown that SGLT2 inhibitors resulted in a gradual recovery and stabilization of renal function after reducing glomerular hyperfiltration in human patients with T1DM (Nespoux & Vallon, 2018; Vallon et al., 2013). However, according to Haneda et al. (2016) SGLT2 inhibitors cause dehydration if water intake is not sufficient, which increases the blood urea nitrogen (BUN) ratio. In our study, kidney function was evaluated through blood tests for BUN, creatinine (CREA), calcium (Ca), phosphorus (P) and symmetric dimethylarginine (SDMA). Although this study did not reveal any significant changes at re‐evaluation after 8 weeks and 12 months, hydration and increased BUN should be monitored in patients with diabetes receiving SGLT2 inhibitors.

SGLT2 inhibitors also have a marked effect on lipid metabolism, acting at different cellular levels (Rhee et al., 2022). It changes the body composition and results in weight loss by reducing the accumulation of lipids, visceral fat and subcutaneous fat (Wang et al., 2017). SGLT2 also regulates key molecules in lipid synthesis and transport and influences the oxidation of fatty acids. Specifically, it enables utilization of lipids and ketone bodies as substrates (Szekeres et al., 2021). In this study, changes in lipids‐related markers, such as TG and T‐chol, were observed in the blood; however, no significant changes were found in the lipid metabolism‐related levels in either group after 12 months of drug administration.

According to previous studies, SGLT2 inhibitors have the potential to provide renal and cardioprotective benefits to patients with T1DM through the reduction of blood glucose levels with low hypoglycaemia risk, glomerular hyperfiltration, blood pressure, volume overload and weight (Bonaventura et al., 2019; Kario et al., 2018; Nespoux & Vallon, 2018; Vallon et al., 2013). Additional studies are needed to determine whether DWP16001 has potential beneficial effects on the kidneys and heart when administered to dogs with diabetes.

This study has several limitations. First, it is known that diabetes is often associated with various diseases (Coady et al., 2019; Fisher et al., 2020; Mattin et al., 2007); however, the effect of administering SGLT2 inhibitors on these diseases was not addressed in this study. Second, this study has no separate control group and limited blood glucose monitoring. In designing the study, patients who had stable blood glucose levels for more than 1 month were selected. Except for cases where drugs that affect blood glucose levels other than insulin were administered, fasting blood glucose and fructosamine levels were measured to observe changes before and after dwp16001 administration. However, it is believed that more detailed conclusions could have been reached if a control group and continuous blood glucose monitoring had been added to the study. Third, in this study, the number of patients who consented to participate in long‐term clinical trial for 1 year was small. Small sample size makes it difficult to check whether the assumptions of the statistical test are satisfied as well as affects the reliability of the results of the subsequent statistical analysis. To compensate for these limitations, future research needs to be conducted by further increasing the population, it is necessary to conduct further clinical trials with more patients. Fourth, the reason why insulin requirements decreased more in the SID group than in the TOD group is presumed to be that the fructosamine concentration decreased relatively in the SID group, resulting in a decrease in insulin requirements, but the cause was not clearly identified. Fifth, both groups showed a decrease in insulin requirements considering the 1‐year average, but at 12 months, the SID group showed an increase in insulin requirements. According to previous studies, continuous exogenous insulin treatment induces the production of insulin antibodies and increases insulin resistance (Hu & Chen, 2018), which may have increased insulin requirements. However, the possibility that the increase in insulin requirements is a temporary phenomenon cannot be ruled out. Therefore, additional long‐term monitoring studies are needed. Nevertheless, this study is meaningful in that it confirmed adverse effects and changes in insulin administered for blood glucose control after SGLT2 inhibitor administration in dogs with naturally occurring type 1 diabetes. This may serve as a reference for future application of SGLT2 inhibitors in insulin‐dependent diabetic dogs.

## 5 CONCLUSIONS

When DWP16001, an SGLT2 inhibitor, was supplied to dogs with type 1 diabetes, no adverse effects were observed, and it was confirmed that the administered insulin dose can be reduced in controlling blood glucose. Although more long‐term randomized trials are needed to determine the long‐term effects of SGLT2 inhibitors as adjuvant therapy in the treatment of diabetic dogs, this study will serve as the reference for the application of SGLT2 inhibitors in dogs with T1DM.

## AUTHOR CONTRIBUTIONS

*Conceptualization; investigation; resources; writing—original draft*: Ju‐Hyun AN. *Investigation; resources; writing—original draft*: Han‐Sol CHOI. *Investigation; validation; writing—review and editing*: Ji‐Soo CHOI. *Investigation; validation; writing—review and editing*: Hyun‐Woo LIM. *Investigation; validation; writing—review and editing*: Wan HUH.*Investigation; validation; writing*—*review and editing*: Ye‐In OH; Joon Seok PARK; Jumi HAN.*Investigation; validation; writing—review and editing*: Soo LIM. *Investigation; validation; writing—review and editing*: Chae‐Young LIM. *Investigation; validation; writing—review and editing*: Tae‐Hee KIM; *Investigation; validation; review*: Jae‐Bong MOON. *Investigation; validation; writing—review and editing*: Hwa‐Young YOUN.

## CONFLICT OF INTEREST STATEMENT

The authors and Daewoong Pharmaceutical Co. Ltd., which supplied DWP16001, declare no conflicts of interest.

### ETHICS STATEMENT

All animal experiments in this study were approved by the institutional animal care and use committee of Seoul National University (SNU), Republic of Korea; all protocol were in accordance with the approved guidelines (SNU; protocol no. SNU‐200921‐5). This experiment was a study on client‐owned animals and was conducted with the prior consent of the owner.

### PEER REVIEW

The peer review history for this article is available at https://publons.com/publon/10.1002/vms3.1454.

## Supporting information



Supplementary data 1. Inclusion and exclusion criteria in this study.

## Data Availability

The data that support the findings of this study are openly available in a public repository that issues datasets with DOIs.
