# Supplementary material for: Effect of the sodium‐glucose cotransporter‐2 inhibitor, DWP16001, as an add‐on therapy to insulin for diabetic dogs: A pilot study
Source: Vet Med Sci. 2024 Apr 30;10(3):e1454. doi: 10.1002/vms3.1454 (PMC11058604; doi:10.1002/vms3.1454)
Supplement: Supplementary file 1 — Supplementary data 1. Inclusion and exclusion criteria in this study. [file VMS3-10-e1454-s001.docx]

| Inclusion criteria (Dogs participating in the test must meet all of the following conditions) |
| --- |
|  Dogs receiving insulin for diabetes.   Prescribed insulin dose must not have changed for at least one month at the start of the test.   Small dogs (<10 kg) under 16 years of age.   Medium dogs (11–22 kg) under 13 years of age.   Nadir blood glucose level of at least 200 mg/dL.   Blood ketone levels of < 0.6 mmol/L.   Dogs that have no trouble administering medication.   Dogs whose owners have given written consent to participate in clinical trials. |
| Exclusion criteria (Dogs participating in the test must not meet any of the following conditions) |
|  Dogs with kidney function problems or related diseases (CKD IRIS stage > 2)   If you receive a rating below average based on BCS (Body Condition Score <3/9).   Dog evaluated for hypotension.   Dogs receiving diuretics or related medications.   Dogs confirmed to be pregnant through a pregnancy test.   Dogs confirmed to be lactating.   Dogs who have taken prescription drugs or over-the-counter drugs within 14 days before the start of the test.   Dogs judged by the clinical investigator to be unsuitable for participation in clinical trials. |

**Supplementary data 1. Inclusion and exclusion criteria in this study.**
